# Editorial: Innate Immune Cells in the Control of Intracellular Bacteria

**DOI:** 10.3389/fcimb.2022.830203

**Published:** 2022-02-03

**Authors:** Luciana Balboa, Andres Baena, Leandro J. Carreño

**Affiliations:** ^1^ Instituto de Medicina Experimental, Consejo Nacional de Investigaciones Científicas y Técnicas (CONICET)-Academia Nacional de Medicina, Buenos Aires, Argentina; ^2^ Grupo de Inmunología Celular e Inmunogenética, Facultad de Medicina, Universidad de Antioquia, Medellín, Colombia; ^3^ Millennium Institute on Immunology and Immunotherapy, Programa de Inmunología, Instituto de Ciencias Biomédicas, Facultad de Medicina, Universidad de Chile, Santiago, Chile

**Keywords:** innate immune cells, intracellular bacteria, pathogens, innate lymphoid cells (ILCs), natural killer T (NKT) cells

Although the great advances in hygiene and health conditions, as well as vaccines against infectious diseases, bacterial infections remain a public health problem, especially in poor and underdeveloped countries. vaccines remain and probably will continue to be the most effective way to prevent and control infection diseases, thus redefining the specificities of the immune responses for each individual pathogen is of key importance for the rational design of novel and more effective vaccines. Although the ultimate goal is to produce robust and long-lasting (memory) adaptive immune responses, to achieve this goal is important to pay attention to the key components that initiate all immune responses: innate immune cells.

Cells of the innate immune system are key players at initiating and regulating adaptive immune responses, impacting the outcome of the control of the infection and the later memory against pathogens. Although classically these cells were thought to be involved in early immune response to infection, it has been described that they can play roles beyond their mainly described effector functions, by modulating the activation and differentiation of the cells responsible for the establishment of adaptive immune response: T cells and B cells. Due to their rapid action and the great influence, they can exert in the outcome of an anti-pathogen immune response, innate immune cells such as natural killer (NK) cells, dendritic cells (DCs), macrophages, innate lymphoid cells (ILCs), natural killer T (NKT) cells, among others, are attractive immunotherapeutic targets to boost immunity against pathogens, as well as to improve vaccine quality. The role that innate cells play in different infections can also be responsible for an immune exacerbation that lead to detrimental inflammation to the host with poor or no elimination of the pathogen. In a world facing the worst pandemic of the century, the understanding of the innate immune response has gained a lot of attention since they can give us several clues to develop immunotherapeutic approaches against pathogen infection. Since the discovery of ILCs in the last decade, a lot of attention has been given to the specific role of innate immune cells in different immune related diseases, with a special emphasis on infection diseases. Determining the specific role that they play in specific infections has taken special importance to design effective vaccines and immunotherapies. This Research Topic aims to shed light on our current understanding of the role of innate immune cells on pathogen infection, focusing on the immune response to intracellular bacteria.

An original research article by Talaei et al. provide a mathematical model to predict the cytokine levels in response to intracellular bacteria, using the facultative intracellular bacteria *Staphylococcus aureus* as a model. In this study, the authors were able to predict and corroborate the levels of TNF-α, IL-6, IL-8, and IL-10 in response to *S. aureus*, demonstrating the potential of this model. Further validation of this model can be very useful to determine the cytokine responses in other bacteria and together with cellular-cytokine interaction models, provide a rational design of immune interventions based on innate immune cells, such as NK cells, NKT cells, macrophages, and ILCs. In line with the importance of the role of NKT cells, Vogt and Mattner provide a very comprehensive review of the contribution of these cells to bacterial infections, giving a detailed description of the lipid antigens recognized by these cells that are present in different bacteria as well as in fungi and some protozoan parasites. They provide a detailed model of activation of NKT cells to boost the control of bacterial infection, either when bacteria provide the NKT-activating lipids, as the case of *Sphingomonas*, *Borrelia*, *Streptococcus*, *Mycobacterium*, *Helicobacter*, *Corynebacterium* and *Bacteroides*, or when bacteria do not possess those ligands and stimulate NKT cell activation *via* PRR recognition and self-lipid recognition. In addition, two review articles discuss the contribution of ILCs to the control and pathogenesis of bacterial infection. Mousaad Elemam et al. described in a very detailed way, the functions of ILCs and NK cells in the control of bacterial infections, as well as the dysfunctional role that they can play in bacterial pathogenesis and immune evasion. They provide an updated view of the roles of NK cells, ILC1s, ILC2s and ILC3s in different infections and the consequences of their dysregulation in bacterial pathogenesis, encouraging novel therapeutic interventions targeting those cells. Finally, Korchagina et al. also provided a review about the role of ILCs in the control of intracellular bacterial pathogens, including a very comprehensive case to case update on their participation in the infection by *Toxoplasma gondii*, *Salmonella typhimurium*, *Yersinia enterocolitica*, *Chlamydia*, *Mycobacterium tuberculosis*, and *Campylobacter*. Finally, the review by Sireci et al. focused on the Q fever-causing intracellular bacterial pathogen *Coxiella burnetiid*, discussing the innate immune cells involved in its control and pathogenesis and coming up with an experimental model to boost immunity against this pathogen based on the complementation of vaccines with the activation of innate immunity by TLR tri-agonists.

The manuscripts included in this Research Topic underline and widen our understanding of the role of innate immune cells in intracellular bacterial infections, highlighting their role in both infection clearance and pathogenesis and their potential use for developing new vaccination strategies aimed to target and modulate innate immune cells.

## Author Contributions

LC, LB and AB edited the topic. LC wrote the manuscript. LB and AB reviewed and corrected the manuscript.

## Funding

Funded by Millennium Institute on Immunology and Immunotherapy P09/016-F (to LC), FONDECYT grant 1211959 (to LC), ICGEB grant CRP-CHL17-06-EC (to LC), COPEC-UC grant 2017.J.924 (to LC), Argentinean National Agency of Promotion of Science and Technology (PICT-2019-01044 to LB) and the Argentinean National Council of Scientific and Technical Investigations (CONICET, PIP 11220200100299CO to LB), Minciencias 111584467121 CT 393-2020 (to AB).

## Conflict of Interest

The authors declare that the research was conducted in the absence of any commercial or financial relationships that could be construed as a potential conflict of interest.

## Publisher’s Note

All claims expressed in this article are solely those of the authors and do not necessarily represent those of their affiliated organizations, or those of the publisher, the editors and the reviewers. Any product that may be evaluated in this article, or claim that may be made by its manufacturer, is not guaranteed or endorsed by the publisher.

